# Applying a User-Centered Approach to Building a Mobile Personal Health Record App: Development and Usability Study

**DOI:** 10.2196/13194

**Published:** 2019-07-05

**Authors:** Leming Zhou, Dilhari DeAlmeida, Bambang Parmanto

**Affiliations:** 1 Department of Health Information Management University of Pittsburgh Pittsburgh, PA United States

**Keywords:** mobile app, personal health record, needs assessment

## Abstract

**Background:**

A personal health record (PHR) system encourages patients to engage with their own health care by giving them the ability to manage and keep track of their own health data. Of the numerous PHR systems available in the market, many are Web-based patient portals and a few are mobile apps. They have mainly been created by hospitals and electronic health record (EHR) vendors. One major limitation of these hospital-created PHR systems is that patients can only view specific health data extracted from their EHR. Patients do not have the freedom to add important personal health data they collect in their daily lives into their PHR. Therefore, there is an information gap between clinical visits.

**Objective:**

The aim of this study was to develop and evaluate a new mobile PHR app that can be easily used to manage various types of personal health data to fill the information gap.

**Methods:**

A user-centered approach was used to guide the development and evaluation of the new mobile PHR app. There were three steps in this study: needs assessment, app design and development, and conducting a usability study. First, a large-scale questionnaire study was conducted with the general population to gain an understanding of their needs and expectations with regard to a mobile PHR app. A mobile PHR app for personal medical data tracking and management was then created based on the results of the questionnaire study. End users were actively involved in all stages of the app development. Finally, a usability study was performed with participants to evaluate the usability of the mobile PHR app, which involved asking participants to finish a set of tasks and to respond to a usability questionnaire.

**Results:**

In the questionnaire study for needs assessment, there were 609 participants in total. The answers from these participants revealed that they wanted to manage various types of personal health data in a mobile PHR app. Participants also reported some features they desired to have in the app. On the basis of the needs assessment findings, a new mobile PHR app (PittPHR) was created with 6 major modules: health records, history, trackers, contacts, appointments, and resources. This app allows users to customize the trackers according to their needs. In the usability study, there were 15 participants. The usability study participants expressed satisfaction with the app and provided comments and suggestions for further development.

**Conclusions:**

This new mobile PHR app provides options for users to manage a wide range of personal health data conveniently in one place. The app fills the information gap between clinical visits. The study results indicated that this new mobile PHR app meets the need of users and that users welcome this app.

## Introduction

### Background

To improve the quality of health care and reduce costs, the Institute of Medicine has recommended the creation of high-quality health data collection systems [1]. This recommendation and the Health Information Technology for Economic and Clinical Health (HITECH) act have led to a dramatic increase in the adoption of electronic health record (EHR) systems in the United States in recent years (from 20.8% in 2004 to 85.9% in 2017), which makes it possible for physicians to easily access detailed patient data [2].

However, the current EHR systems only store data collected during patients’ clinic and hospital visits. Anything happening in between those visits is not included in EHR systems, for instance, did the patient take prescribed medication on time? Did the patient become more active after the doctor suggested increasing physical activity? To what extent did the mood of the patient stabilize in the period of time after a mental health intervention was delivered? This type of information can be critically important for health care providers to determine the effectiveness of their treatment strategy and to identify the reason behind disappointing treatment outcomes. In most cases, health care providers can only obtain this type of information by asking their patients when they visit a clinic or hospital, and the information obtained at that moment is typically not highly reliable because the accuracy of the data mainly depends on the memory of the patient.

To fill this *information gap* and to empower patients so that they can be more active in their own health care, one approach is to provide personal health record (PHR) systems to patients and allow patients to access and manage their own health data [3-6]. According to the Office of the National Coordinator for Health Information Technology (ONC), PHR is “an electronic application through which patients can maintain and manage their health information (and that of others for whom they are authorized) in a private, secure, and confidential environment” [7]. The International Organization for Standardization (ISO) also provided a definition for PHR and emphasized the health records in PHR should be “primarily managed and controlled by the individuals who is the subject of the record, or his/her authorized representative” [8].

### Previous Work

There are many PHR systems on the market; many are Web portals created by hospitals or EHR vendors to provide patients *preselected* data items from corresponding EHR systems, such as laboratory test results, medications, immunization records, visit notes, and appointment schedules [9]. This type of PHR is called by many different names, such as EHR-tethered PHR portal, tethered PHR portal, PHR portal, or simply, patient portal. One example of a PHR portal is My HealtheVet from the Veterans Health Administration [10,11]. Another example is MyChart*,* created by Epic Systems Corporation and used by many hospitals. Both MyChart and My HealtheVet provide patient data access, prescription refills, and *a few* patient-reported health data items, such as blood pressure and blood sugar. Several other Web-based PHR portals offer similar major features, with variations in terms of the patient information tracking tools, specific data items provided, and patient populations targeted [12-17].

These PHR portals provide useful health-related data access to patients and empower patient self-management. Some earlier studies reported improved quality of care and clinical outcomes as a result of using PHR portals [17-19]. The common limitation of these PHR portals is that they do not provide much flexibility to patients in terms of what health data generated between clinical visits can be entered. In other words, the *information gap* mentioned earlier is still there, even with the availability and use of these PHR portals.

In recent years, PHR technology has moved forward, and a number of mobile PHR apps have been created, such as the mobile app version of MyChart, an EHR-tethered PHR app named MyHealthKeeper, and several mobile PHR apps without unique app names [20-22]. These mobile apps make access to PHRs easy, given the rapid growth of mobile device ownership in recent years and the high portability of mobile devices.

However, most of these mobile PHR apps are also limited, as they only offer features similar to those in Web-based PHR portals. Moreover, many of them were also created by hospitals and EHR vendors, meaning the major data source for these mobile PHRs was still the corresponding EHR systems. Hence, although these mobile PHR apps can provide a certain level of convenience and empowerment to patients, they still cannot fill the *information gap* between the typical clinical visits because patients can only enter very limited types of personal health data items in these PHR mobile apps [6,23]. In other words, as with those PHR portals, the patient data are still fully controlled by hospitals and health care providers; therefore, patients can only use these PHR systems to *access and manage data items selected by health care providers*.

This could be one major reason for high interest in but low adoption of PHR portals and mobile PHR apps [24,25]. In several previous research studies and systematic reviews, study participants expressed the belief that PHRs could be useful for better quality of health care; however, before they actually use any PHR portals or apps, they expect PHRs to offer a wide range of secure, user-friendly, and patient-centered functionalities so that they can perform self-management of their conditions [6,19,26-30]. Examples of desired features are personalization for patients and their health issues and patient-generated health data (PGHD) reporting [31]. This study will mainly focus on the PGHD reporting as the availability of this feature will allow the information gap between patients’ clinical visits to be filled.

According to the definition from the ONC, PGHD is “health-related data created and recorded by or from patients outside of the clinical setting to help address a health concern” [32]. One type of PGHD is readings from various wearable sensors. In recent years, many types of wearable sensors and their corresponding mobile apps have been released to the market. A number of research studies have evaluated the reliability of these wearable sensors. The study results have indicated that some wearable sensors are highly reliable in terms of some health data tracking such as steps, heart rate, and sleep [33-39]. Hence, the data items from these wearable sensors are sufficiently accurate to make them valuable for patients’ health management and monitoring. Many people have started to use these wearable sensors to track their health data [40-42]. However, the data collected from these wearable sensors are currently stored in different places such as the memory of devices or the corresponding mobile apps, which makes it difficult for patients to manage these data items.

Then, a desirable feature of PHR portals or apps would be the ability for patients to store readings from these wearable sensors in *one single place* so that they can manage *all* of their health data conveniently [6,23]. It is believed that if all patient-needed health data are stored in a PHR portal or mobile app, the PHR adoption rate might improve because the PHR could satisfy patients’ health information needs and further improve the convenience of using it. Their health data would become easier to manage. Moreover, reliability of the health data could also be improved because patients only need to manage one copy of their health records. This single place storage for all health data also would make it easy for patients to share their health data with their health care providers, which in turn might encourage health care providers to utilize patient-generated data in their clinical decision making [6,23]. Unfortunately, many existing PHR portals and mobile apps do not provide this desired feature.

### Objectives

In this study, a user-centered approach was used to develop and evaluate a new mobile PHR app. This mobile app can be used to manage various types of personal health data, including the data types usually offered in the current PHR systems (Web portal or mobile app), as well as the data items generated by multiple types of trackers and personal health monitoring devices such as pedometers, smart watches, digital blood pressure monitors, and digital weight scales. It is expected that this new mobile PHR app is lightweight, highly portable, and convenient to use and will serve the target users better in terms of personal health data management. Here, the target users are anyone who want to manage all their health data in a single place. The app is expected to fill the information gap between clinical visits.

In the remaining of this article, we describe the methods, results, discussion, and conclusions of the study. The Methods section presents the study design and procedure for a needs assessment, the mobile app architecture, and the usability study. The Results section provides the results obtained in the needs assessment, the features implemented in the mobile PHR app according to the assessment, and the outcomes of the usability study. The Discussion section explains the principal findings, comparison with other studies, and the limitations of this study. The last part is the conclusions of the study.

## Methods

### User-Centered Approach for App Development and Evaluation

In a user-centered approach, target users of a system are actively involved in all the stages of system development, including requirement analysis, system design and implementation, and system evaluation. With regard to this project, it includes (1) specifying users’ requirements, (2) designing the app according to users’ requirements, (3) having users evaluate the mobile app (usability study), and (4) making all necessary adjustments to the app design and implementation according to users’ feedback [43,44]. These are the steps we took in this study. The details of these steps are described in the following sections.

### Questionnaire for Needs Assessment

To create the desired mobile PHR app, the first step was to perform requirement analysis by collecting users’ expectations with respect to the specific data items they plan to manage in the app. A questionnaire with 14 questions was created to collect target users’ ideas about a mobile PHR app.

In the first section of the questionnaire, respondents were asked to provide answers to a set of demographic and background questions. The demographic questions were about age, gender, race, marital status, education, and income level. In addition, two background questions were asked about the health status and experience using mobile health (mHealth) apps: (1) What is your own assessment of your health? (2) Have you used mobile health apps before?

In the second section of the questionnaire, 6 questions were used to determine users’ desired health data content and format. For each question, there was a list of options, and the respondents were allowed to choose one option (Q3 and Q5) or multiple applicable options (Q1, Q2, Q4, and Q6) or add their own answers. The options for the first question were arranged in three groups. The following are the 6 questions:

- Q1. What content would you like to see in a mobile PHR app?
  - Q1.1. Medical records to manage
  - Q1.2. Information to track
  - Q1.3. Other information
- Q2. What specific health issues do you plan to manage with this proposed PHR app?
- Q3. What type of user interface works the best for you in the proposed PHR app?
- Q4. If you plan to manage your laboratory test results in this proposed PHR app, what is the desired format for showing the laboratory results?
- Q5. Do you expect to see an overview dashboard to show the summary of your health information in this proposed PHR app?
- Q6. What type of security protection do you expect to see in this proposed PHR app to protect your personal health information?

This questionnaire study was conducted via the Web-based Qualtrics system (Qualtrics). A public announcement including the purpose of the study and the link to the questionnaire was distributed to roughly 2000 recipients via a bulk email system at the University of Pittsburgh. Study participants provided their answers to these questions on the Web-based system. The obtained data were statistically analyzed using SPSS version 25 (IBM).

### Mobile App Design and Development

A mobile PHR app (PittPHR) was designed and developed based on the information collected in the questionnaire study. Users were involved throughout the PHR app’s design and implementation process. During both the design and implementation stages, users actively contributed their ideas and provided their feedback to multiple versions of the app prototype in terms of usability and user experience. Their suggestions have been incorporated into the current version of the mobile PHR app.

The Ionic 3 (Drifty, Co.) was chosen to implement this mobile app as it is a cross-platform framework allowing the app to run on mobile devices with iOS, Android, or Windows Phone system. The app can be deployed natively on a mobile device using Apache Cordova (Apache Software Foundation). After the deployment, the app runs in the Web browser as a Progressive Web App. The app cannot be used if an internet connection is not available. PostgreSQL (PostgreSQL Global Development Group) was chosen for the back-end database because it is a powerful, highly scalable, cross-platform, and free open-source relational database system.

**Figure 1:**
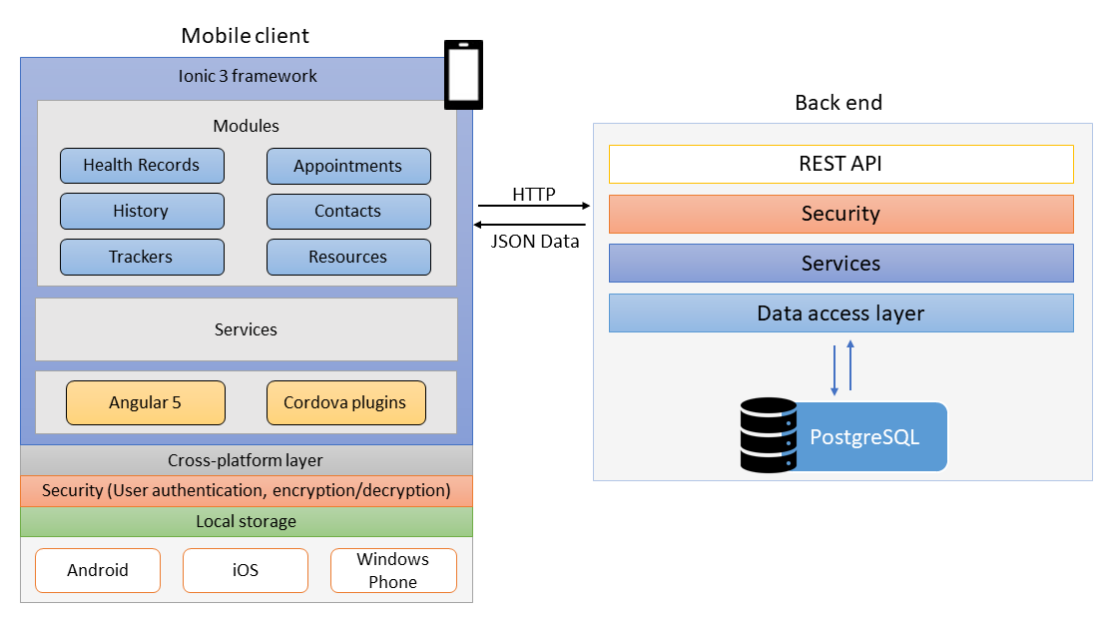
System architecture of the mobile personal health record app. REST: REpresentational State Transfer; HTTP: HyperText Transfer Protocol; API: application programming interface; JSON: JavaScript Object Notation.

Figure 1 shows the architecture of the app. The left-hand side is the mobile client app running on the user’s mobile device, including the modules identified in the needs assessment study, supported by Angular 5, Cordova plugins, and security services (mainly user authentication, encryption, and decryption). Local storage is available for this system but is not used for any patient data storage. On the right-hand side are the components of the back-end server, mainly the PostgreSQL database, which can be accessed by the mobile client app via the interface of the REST (REpresentational State Transfer) API (Application Programming Interface). The data exchange between the mobile client and the back-end server takes place through the HTTP protocol and the JSON (JavaScript Object Notation) data standard.

Data encryption and user authentication features are implemented in the app for patient data security protection. When a user has a specific data item to enter into this mobile app, the user needs to log into the mobile PHR app (user authentication), choose a specific category, and enter the information into the app. The entered data are encrypted with the Advanced Encryption Standard (AES) algorithm and sent to the remote secure server behind a firewall for permanent storage. The entered data are only stored on the remote server in an encrypted format. To review health records on the mobile app, the encrypted data are retrieved from the remote server and decrypted only on the mobile device to show the content. In other words, only authenticated users can enter and view their own health data on the app.

### Mobile App Evaluation

A usability study was conducted on PittPHR with 15 participants to evaluate the usability of the mobile PHR app. In a usability study, 4 to 5 participants are sufficient for identifying 80% of usability issues, whereas 15 participants are sufficient for identifying all usability problems [45-47]. The study advertisement was posted on the Pitt + Me website [48]. Potential study participants expressed their interest in participating in this usability study on the website, and a random selection was performed from this potential participant pool by considering their age (≥18 years and having representatives of different age groups) and gender (balanced male and female participants). The study protocol was approved by the institutional review board at the University of Pittsburgh.

In this usability study, after signing a consent form, the study participants were introduced to the PittPHR app by being given the purpose of the study and a brief demonstration of the features in the app. The study participants were then required to complete several tasks, including (1) logging into the app on a mobile device (iPad Mini 4, iOS 11.4, 7.9-inch); (2) entering records in various categories such as laboratory test results, immunization records, medical history, allergies, food consumed in one day, and a doctor’s appointment; (3) reviewing the entered health information; and (4) updating the information on the app. Both the mobile device and the health-related records entered into the mobile app were provided by the research team.

Upon completion of all assigned tasks, the participants were asked to complete the Post-Study System Usability Questionnaire (PSSUQ) to provide their overall impression of the app [49]. The study participants were also asked to provide general comments and suggestions regarding the mobile PHR app after they filled out the usability questionnaire. Descriptive statistics of the responses to the usability questionnaire were calculated using SPSS version 25. The participants’ comments were summarized.

## Results

In this study, we performed the needs assessment using a questionnaire to collect ideas directly from users of the app, implemented the user-desired features in the new mobile PHR app, and evaluated the usability of the app. This mobile PHR app enabled users to manage all their health data conveniently in one place, which in turn may encourage users to be more involved in their health care and fill the information gap between typical clinical visits.

### Results of the Questionnaire Study

In total, 609 people answered the Web-based questionnaire. As the email announcement about the questionnaire was distributed to approximately 2000 people, the response rate was approximately 30.45%. Considering that the email announcement was only distributed once, this response rate was good. The demographic information for these respondents and their answers to the background questions (self-assessed health status and experience of using mHealth apps) are summarized in Table 1. The numbers show that these study participants consist of people of different age groups, genders, races, education levels, marital status, income levels, mHealth app use experiences, and health status. For each question, there were a few to several subjects who chose not to provide an answer to the question. These were different people for different questions. Those numbers are not shown in the table. Please note that for the race question, *other* was one of the options; similarly, for the household income question, *decline to answer* was one of the options. Therefore, the corresponding numbers are listed in the table.

The answers to the questions about user-desired data contents, format, and other features in a mobile PHR app are summarized in Table 2. As mentioned in the Methods section, when the study participants provided their answers to Q1, Q2, Q4, and Q6, they were allowed to choose one or multiple options and add answers that were not shown in the given options. They were only allowed to choose one option or add their own answer to Q3 and Q5. Again, there were some study participants who chose not to answer some questions.

The answers to Q1 are arranged into three groups in Table 2, that is, medical records to manage, information to track, and other desired information. The data in the *medical records to manage* group are the typical medical records such as laboratory test results, doctor visit notes, medication, immunizations, medical history, and social history. More than half of the study participants indicated that they would like to use the mobile PHR app to manage the first 5 types of data items. Only 11.5% of the study participants wanted to use the app to manage social history. Some respondents suggested including additional types of records such as surgical history, family history, and allergies.

In the *information to track* group, the respondents chose many types of data that they wanted to track in the app. In Table 2, only a few major ones are listed, including nutrition, physical activity, health activity, and health diary. Other data items specifically mentioned by the study participants in their answers as items they would like to track are calorie intake, weight, pain level, period, lens prescription, and sleep duration.

In the *other information* group, the respondents indicated multiple types of other data items that they would like to see in the mobile PHR app, such as reliable resources for various diseases, family members’ and doctors’ contact information, and doctor’s appointments.

The study participants’ answers to Q2 provided the specific health issues they would like to use the mobile PHR app to manage. The top 4 were weight management, medication management, cardiovascular disease, and diabetes. Some respondents (116/609, 19.0%) mentioned several other health issues they would like to manage with the mobile PHR app, such as pain, sleep, asthma, blood pressure, anxiety, and stress.

In the answers to Q3, the study participants indicated their preferences regarding the user interface of the mobile PHR app. More than 40% of the study participants claimed that they would like to see a colorful and graphical user interface. Approximately 31% of the study participants (188/609, 30.9%) reported that they would like to have a customizable user interface, and around 21% (127/609, 20.9%) stated that they would like to have a text-based user interface.

In Q4, the study participants reported their preferred format for laboratory results: 40% (243/609, 39.9%) of the study participants preferred to have their laboratory results shown as lists, close to 40% liked tables, and around 20% liked to see them as graphs.

The study participants were asked whether they needed an overview dashboard in Q5. About 60% of them claimed that they would like to have an overview dashboard in the app to quickly check the recently updated health data on a single page. Close to 30% of them were not sure whether a dashboard was necessary, and a small number (38/609, 6.2%) felt that it was not necessary.

**Table 1:** Demographics and background of the study participants (N=609).

Demographics		Value
**Age (years), mean (SD)**		43.3 (13.28)
	18-28, n (%)	106 (17.4)
	29-45, n (%)	222 (36.5)
	46-55, n (%)	142 (23.3)
	≥56, n (%)	131 (21.5)
**Gender, n (%)**		
	Male	134 (22.0)
	Female	473 (77.7)
**Race, n (%)**		
	African American	15 (2.5)
	White	557 (91.5)
	Asian	21 (3.4)
	Other	13 (2.1)
**Education, n (%)**		
	High school or lower	15 (2.5)
	Some college credits, no degree	55 (9.0)
	Associate degree	31 (5.1)
	Bachelor’s degree	231 (37.9)
	Master’s degree	176 (28.9)
	Doctoral degree	89 (14.6)
	Professional degree	10 (1.6)
**Marital status, n (%)**		
	Single	157 (25.8)
	Married	406 (66.7)
	Divorced	37 (6.1)
	Widowed	6 (1.0)
**Household income, n (%)**		
	< US $25,000	24 (3.9)
	US $25,001-US $50,000	109 (17.9)
	US $50,001-US $75,000	109 (17.9)
	US $75,001-US $100,000	96 (15.8)
	US $100,001-US $125,000	71 (11.7)
	>US $125,000	116 (19.0)
	Decline to answer	70 (11.5)
**Used mobile health apps before, n (%)**		
	Yes	359 (58.9)
	No	248 (40.7)
**Self-assessed health status, n (%)**		
	Excellent	53 (8.7)
	Very Good	263 (43.2)
	Good	234 (38.4)
	Fair	55 (9.0)
	Poor	3 (0.5)

**Table 2:** Summary of answers to questions about data content and format and desired features (N=609).

Contents and features		Value, n (%)
**Q1.1. Medical records to manage**		
	Test results	398 (65.4)
	Doctor visit notes	272 (44.7)
	Medication	372 (61.1)
	Immunizations	382 (62.7)
	Medical history	389 (63.9)
	Social history	70 (11.5)
**Q1.2. Information to track**		
	Nutrition	417 (68.5)
	Physical activity	413 (67.8)
	Health activity	371 (60.9)
	Health diary	295 (48.4)
**Q1.3. Other information**		
	Reliable resources	268 (44.0)
	Other	46 (7.6)
**Q2. Health issues to manage**		
	Weight management	428 (70.3)
	Medication management	209 (34.3)
	Cardiovascular disease	89 (14.6)
	Diabetes	43 (7.1)
	Other	116 (19.0)
**Q3. User interface**		
	Colorful and graphical	259 (42.5)
	Customizable user interface	188 (30.9)
	Clean text interface	127 (20.9)
**Q4. Format for laboratory results**		
	List	243 (39.9)
	Table	237 (38.9)
	Graph	131 (21.5)
**Q5. Overview dashboard**		
	Yes	368 (60.4)
	Maybe	173 (28.4)
	No	38 (6.2)
**Q6. Security protection**		
	User authentication	503 (82.6)
	Encryption	230 (37.8)
	Data backup	131 (21.5)

The last question, Q6, was about user-desired security protection. The vast majority of the study participants indicated that they wanted to see user authentication in the mobile PHR app. Other desired security protection features listed were data encryption and data backup. Some study participants even further indicated the user authentication methods, such as biometrics.

### Implemented User-Desired Features of the New Mobile Personal Health Record App

The user-desired contents and features identified in the questionnaire study were implemented in the mobile PHR app (PittPHR). As shown in Figure 1, there are 6 major modules in this mobile app: Health Records, History, Trackers, Appointments, Contacts, and Resources. The *Health Records* module is used to manage frequently updated medical information, such as laboratory test and diagnostic test results, doctor visit notes, medications, and immunization records. The *History* module is used to manage relatively stable medical data, such as medical history, family history, surgical history, allergies, and social history. Users can use the *Tracker* module to track 12 different types of personal heath data, such as weight, blood pressure, physical activity, food, drink, sleep, period, and pain. The *Appointments*, *Contacts*, and *Resources* modules are used to manage all types of doctor’s appointments, contacts, and links to health resources. Both the *Trackers* and *Resources* modules are customizable; users can customize the trackers according to their own needs by hiding or unhiding available trackers in a given list, and they can add or delete links in the Resources module according to their own needs. All 6 modules are supported by the cross-platform layer; therefore, they can run on all three major mobile operating systems (Android, iOS, and Windows Phone system). Data security protection features were implemented in the app to conduct user authentication and data encryption and decryption.

**Figure 2:**
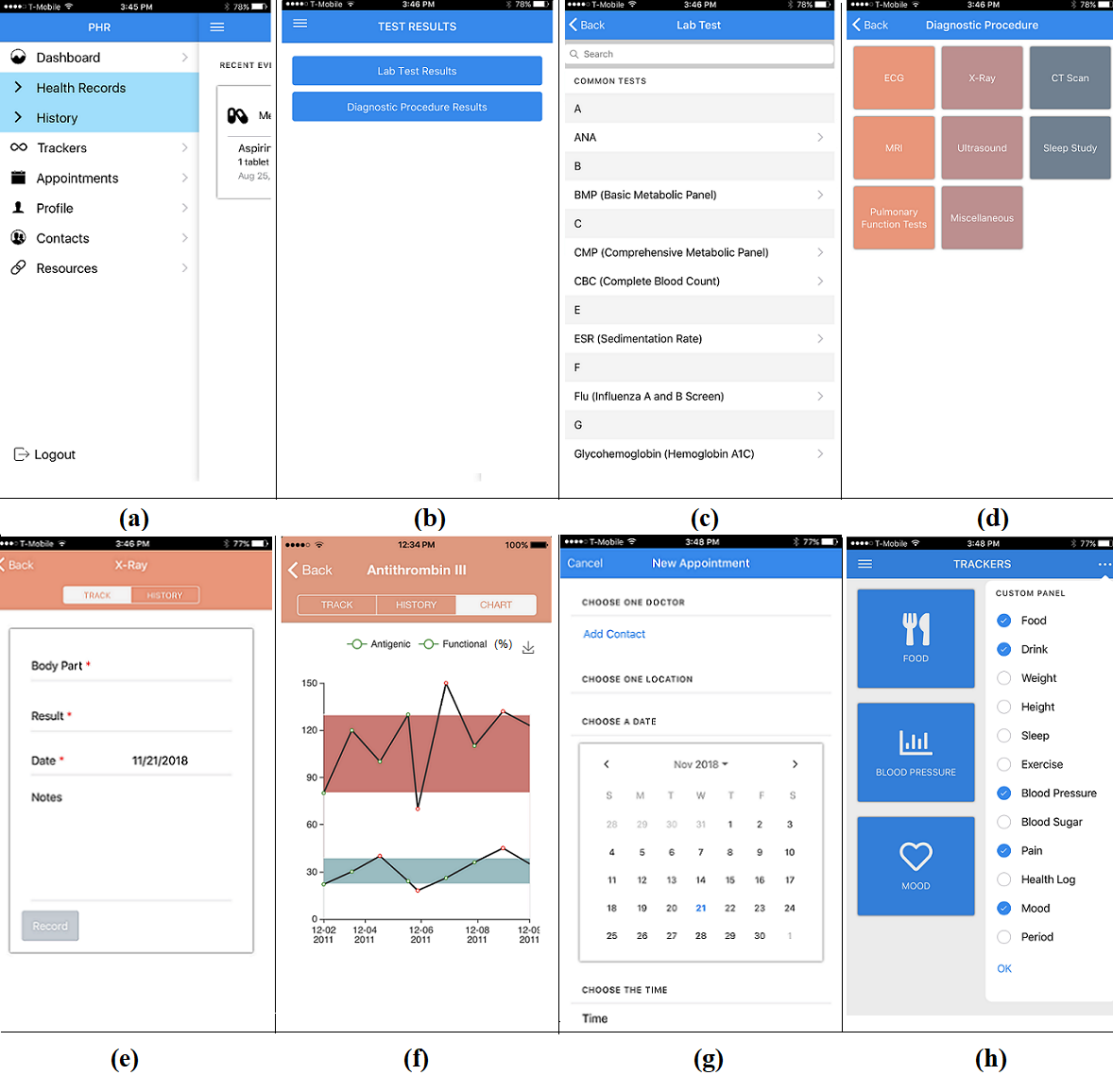
Screenshots of the new mobile personal health record app. (a) Dashboard. (b) Two types of tests in the Test Results section under the Health Records module. (c) A list of commonly ordered laboratory tests. (d) A list of buttons for commonly ordered diagnostic procedures. (e) A simple form for entering a typical x-ray exam result. (f) Graphical test results collected over time. (g) The page for adding a doctor’s appointment. (h) The customizable tracker choices.

The users’ preferences on data content and format were incorporated in the new mobile PHR app. Figure 2 shows eight screenshots of the mobile PHR app. The first 4 screenshots on the top will be described from left to right (a-d), and then the next 4 screenshots on the bottom will be described from left to right (e-h). This mobile app has a colorful and graphical user interface. The first screenshot (top left) in Figure 2 displays a list of modules in the app on the left-hand side and the contents of the dashboard (truncated) on the right-hand side. The dashboard shows a summary of recently entered data, for instance, a new medication—aspirin. The Health Records and the History modules can be expanded to show further details. The second screenshot in Figure 2 shows the screen that appear if the Test Results section in the Health Records module is selected. The third and fourth screenshots in Figure 2 display the screens that appear when each of the two buttons in the second screenshot is selected. The third screenshot has a list of commonly ordered laboratory tests, organized in alphabetical order. The fourth screenshot (top right) includes a list of commonly orders diagnostic procedures.

If any of these test items is clicked, a very brief form will be shown under the tab TRACK in the fifth screenshot (bottom left) in Figure 2 so that the app user can enter data items such as the date, test result (numbers or texts), and any additional notes the user wants to enter. The test results entered over time will be shown as a list under the tab HISTORY, shown in the fifth and sixth screenshots. If the test results are quantitative, the history can also be shown as curves under the tab CHART, as shown in the sixth screenshot. In other words, some quantitative test results can be shown either as a list or a graph according to the user’s preference. For some tests, the normal range of test values has been incorporated into the app and can be shown as a shadowed band or a straight line in the chart. If the normal range of test results is not available or has not be incorporated into the app, only the test results will be shown in the chart.

The seventh screenshot in Figure 2 displays the page for adding a new doctor’s appointment. If the doctor’s contact information is already stored in the Contacts module in the app, users can simply choose the doctor from the contact list and indicate the appointment date and time in the form and calendar shown in the seventh screenshot. If the chosen doctor has multiple office locations, users also have the option to select the location of that specific appointment. If the doctor’s contact information is not stored in the Contacts module, users can add the contact information by clicking the *Add Contact* button in the seventh screenshot to fill out a form with the doctor’s full name, office addresses, specialty, office phone number, and fax number (not shown).

The last screenshot (bottom right) in Figure 2 shows the page for health data tracker selection. Users can choose their desired trackers from the list on the right-hand side after they click the three dots at the top right corner of the page. Once users make their selection, only the chosen trackers will be shown in this page. To use a tracker, users can click the icon and fill out a brief form for the tracker. For instance, to report the blood pressure, users only need to provide the numbers for the systolic and diastolic readings plus the date and time.

The details of a few other modules are not shown in Figure 2. The Contacts module offers users a space to store several types of contact information, such as emergency contacts, family members, friends, and doctors. The Resources module allows users to manage website links to Web-based resources useful for themselves. There is also a Profile section in the mobile app, which is used to show users’ basic information such as name, address, age, gender, phone number, and email address.

All the modules in this mobile app are designed to be extensible according to the needs of users. For instance, more laboratory tests, diagnostic procedures, and new trackers can be added to this app.

### Usability Study Results

The usability study was performed with 15 participants from June 2018 to October 2018. These participants were selected from among 114 persons who expressed their interest in participating in this usability study on the Pitt + Me website. The demographics of the 15 usability study participants are summarized in Table 3.

Overall, 9 (60.0%) participants used mHealth apps regularly. Frequently mentioned mHealth apps were Apple Health, MyFitnessPal, Samsung Galaxy Health, Map My Ride, Cardiio, and Fitbit. One participant mentioned that she used a calorie intake app, a daily pill reminder, and a period tracker but did not provide specific names of these apps.

**Table 3:** Demographics of the usability study participants (N=15).

Demographics		Value
Age (years), mean (SD)		35.3 (15.24)
**Gender, n (%)**		
	Male	8 (53.3)
	Female	7 (46.7)
**Race, n (%)**		
	African American	2 (13.3)
	White American	8 (53.3)
	Asian American	5 (33.3)
**Education, n (%)**		
	High school or lower	2 (13.3)
	Some college credits, no degree	1 (6.7)
	Bachelor’s degree	3 (20.0)
	Master’s degree	5 (33.3)
	Professional degree	2 (13.3)
	Doctoral degree	2 (13.3)
**Marital status, n (%)**		
	Single	9 (60.0)
	Married	6 (40.0)
Years used smart devices, mean (SD)		7.8 (2.4)
**Used mobile health apps before, n (%)**		
	Yes	13 (86.7)
	No	2 (13.3)
**Self-assessed health status, n (%)**		
	Excellent	4 (26.7)
	Very Good	7 (46.7)
	Good	3 (20.0)
	Fair	1 (6.7)

All study participants were able to finish the given tasks in the usability study easily, in approximately 15 min on average. The details of the study participants’ responses to the PSSUQ statements in this usability study are provided in Table 4. Participants could choose from 1 to 7, where 1 means strongly agree, whereas 7 means strongly disagree. Therefore, the lower (closer to 1) the values of these statements, the higher the usability of the app because lower values indicate that these study participants agreed that the app was easy to learn, was easy to use, was effective to finish tasks, had all desired features, and had a good user interface and that they were satisfied with the app. This is also true for the overall average of the PSSUQ scale, that is, a lower overall average value corresponds to a higher usability of the app. In this study, the overall average of the participants’ response in the PSSUQ was 1.90 (SD 0.526). Therefore, the usability of the app was shown to be high. The small SD indicates that these study participants’ opinions were consistent.

The following are some comments from study participants about their overall impression of the PHR mobile app:

It was easy to use and easy to learn. I want to download the app and use it

Participant 5

I like this app and would like to use it if it were available for download. Overall, the app was easy to understand

Participant 2

It is a handy app to keep lab test results. I like the different color-coded sections.

Participant 9

This app is easy to use and user friendly

Participant 12

This is a pretty good and easy to use system

Participant 15

**Table 4:** Usability study results.

Statements	Mean (SD)
Overall, I am satisfied with how easy it is to use this system.	1.73 (0.704)
It was simple to use this system.	1.67 (0.724)
I could effectively complete the tasks and scenarios using this system.	1.53 (0.516)
I was able to complete the tasks and scenarios quickly using this system.	1.40 (0.507)
I was able to efficiently complete the tasks and scenarios using the system.	1.67 (0.724)
I felt comfortable using this system.	1.53 (0.640)
It was easy to learn to use this system.	1.40 (0.507)
I believe I could become productive quickly using this system.	1.73 (0.704)
The system gave error messages that clearly told me how to fix the problems.	3.47 (0.834)
Whenever I made a mistake using the system, I could recover easily and quickly.	2.40 (1.404)
The information (such as on-line help, on-screen messages and other documentation) provided with this system was clear.	2.67 (1.397)
It was easy to find the information I needed.	1.67 (0.900)
The information provided for the system was easy to understand.	1.67 (0.724)
The information was effective in helping me complete the tasks and scenarios.	1.67 (0.724)
The organization of information on the system screens was clear.	1.60 (0.632)
The interface of this system was pleasant.	2.00 (1.069)
I liked using the interface of this system.	1.97 (0.915)
This system has all the functions and capabilities I expect it to have.	2.40 (1.549)
Overall, I am satisfied with this system.	2.00 (1.254)

In addition to these general comments, study participants also provided specific suggestions for further improvement on the app. We assigned these suggestions to one of 4 common themes that emerged upon analysis. When multiple participants mentioned the same feature, only one representative comment is cited here.

- Theme 1 Connect to other information systems to reduce the data entry load and share the information with others, such as doctors:

Would it be able to pair with sensors and show real-time data?

Participant 6

Can this be connected to the standard health records?

Participant 7

For social, family history etc., can you include “canned” items already, so that we may minimize the amount of data that we need enter?

Participant 8

Can you link this app to the phone’s calendar and load the existing doctor’s appointments?

Participant 14

It would be nice if my doctors could also see the data I entered in the app

Participant 15

Can you connect to the phone’s camera so that I can upload photos?

Participant 17

- Theme 2 Reminders and alerts:

Would there be an alert going out to me when the medication needs to be re-filled?

Participant 12

It would be helpful to have a description for each vaccination so that the user is reminded when to do next ones

Participant 14

It would be helpful to have an alert for upcoming appointments

Participant 18

- Theme 3 Standardization of patient-entered information:

It would be nice to have a section saying what the normal range for each test result is.

Participant 12

If the medication names were from a database, users would not need to know the exact spelling of each medication.

Participant 12

A pre-populated drop-down list whenever possible on all occasions would be good, such as for medication names, test result units, food servings, and diagnosis

Participant 14

- Theme 4 Other desired features for convenience and flexibility:

Can the tracking data be graphed? For example, show the quality of sleep (episodic tracking).

Participant 8

For medication, add a notes section.

Participant 9

It would be helpful to track daily activity and provide on dashboard showing how much calories were consumed each day etc

Participant 14

Can you add QR code capability?

Participant 16

## Discussion

### Principal Findings

In this project, a user-centered approach was used to design, implement, and evaluate the mobile PHR app, PittPHR. This app provides users with the ability to collect and manage all their health data in a single place, including the typical medical records and PGHD. The features of this app may encourage users to be more involved in their own health care and eventually improve health care quality [50-52]. Use of this mobile app to flexibly manage various types of patient health data may also fill the information gap currently existing between clinical visits.

Before designing and developing this app, to determine user’s needs and preferences, a short questionnaire was created and distributed to approximately 2000 recipients. In total, 609 persons answered the questionnaire and indicated desired data items and features in the mobile PHR app. These collected preferences were then used to guide the design and development of the new mobile PHR app. Specifically, 6 app modules for frequently updated health information, records that are not updated so frequently, customizable trackers, contact information management, appointment management, and customizable resource links were implemented into the mobile app. These modules made it convenient for users to manage their health data. A study with 15 participants was performed to evaluate the usability of the app.

For the general population, EHR and PHR are two highly similar systems. Therefore, when they answered the questionnaire for the needs assessment, their desired data items in a PHR are similar to the ones in existing EHRs. One major difference is the desire for managing PGHD between typical clinical visits. The study participants want to use a PHR to manage their PGHD.

The app was designed to be easy to use. The individual pages in the new mobile PHR app were designed to be simple, with each page having only a specific purpose, for instance, entering laboratory test results, viewing the entered data, and making note of an appointment. Therefore, the usability of the app was high (1.90 out of 7 in PSSUQ) and the study participants were satisfied with the app.

This app is customizable and flexible to use for managing data collected from various types of wearable sensors. In the current version of the app, there are 12 trackers, including food, drink, weight, height, sleep, exercise, blood pressure, blood sugar, pain, mood, period, and health notes. Users can choose the trackers according to their needs. As there are many types of wearable sensors and each of them has its own API and this field itself is changing quickly [35,53], it is challenging to integrate all these APIs into this mobile app and keep them updated all the time. Therefore, in the current version of this mobile PHR app, we chose the simplest approach for data collection, providing a list of trackers and asking the user to choose the trackers they use and then enter the data items from those trackers manually [6]. In the future, after the wearable sensor market is mature and APIs used are relatively stable, we can integrate them into this mobile app and make the data collection automated.

This app has strong security measures to protect user data. Although it is not the major focus of this study, user information security and privacy has been a top priority throughout the design process. In this mobile PHR app, users are required to register their own accounts with a strong password (a combination of upper-case and lower-case letters, digits, and special symbols) before they can start to use the app. All user-entered data are encrypted before they are transmitted on the internet and stored on a remote secure server behind a firewall. The user-entered data are decrypted after the app retrieves the data to display on the local device, and only authorized users can enter, view, or change the data in the app. In this design, the mobile PHR app cannot be used when an internet connection is not available. In other words, it requires a Wi-Fi signal or cellular service to work. All the collected data are only stored in an encrypted format on the remote secure server. No health record is stored on the local storage of the mobile device. Therefore, even if the user loses the mobile device, there will not be any health data breaches. The user can continue using the app on a different mobile device without losing any data.

PittPHR is a Web-based app; therefore, the resources required on the mobile device are similar to those for other Web-based apps. The scalability of the app is determined by the capacity of the remote secure server. At this moment, it is a typical Dell server. If the number of users increases to a large number (a few thousand or more), a cloud-based server may be needed. PittPHR can run on any major mobile operating system (iOS, Android, and Windows Phone system). This is one advantage when compared with the *operating system–specific* Health apps offered by Apple and Google. Further details are provided in the next section.

With the availability of this mobile PHR app, users will be able to conveniently manage all of their health data in one place, including the typical medical record data and the patient data generated between their typical clinical visits, data that are often unavailable to medical professionals. As a result, the information gap will be filled, and health care providers may obtain more reliable and comprehensive patient data, which may help them to better understand the reasons for the ineffectiveness of certain therapies. Health care providers can utilize the information in their decision making, which may lead to improvements in the quality of health care provided.

The design and implementation of the app also have integrated some solutions to the PHR adoption barriers. As mentioned in the Introduction section, the adoption rate for PHRs is still low, with barriers to adoption including factors such as the demographic characteristics of users and security and privacy concerns [28,54]. With respect to demographics, women and the older adults tend to actively use PHRs less often [55]. The questionnaire study for needs assessment allowed us to consider this issue as there were more than 100 respondents in each age group and the average age of the 609 participants was 43.3 years. More specifically, we obtained opinions from a large number of people older than 56 years, including 26 participants who were 65 years or older. Moreover, among all of the respondents, 78% (473/609) were female, meaning opinions from women are reflected in the questionnaire study results. We addressed the second barrier to adoption, security and privacy concerns, by including strong security measures in the app. These features may help this new mobile PHR app to achieve a higher adoption rate than others.

This mobile app is designed to be extensible, and therefore, it will be convenient to add new features according to users’ needs and feedback. The intention of this study is not to create a mobile app to meet the needs of everyone but to build the mobile app and provide it to users to use. Once the users have used the app for a period of time, they will have a better idea of what they want. We will collect feedback from these actual users and update the app to make it better [15,20,56,57], changing existing features and adding new features into the app, for instance, creating more types of trackers, adding more laboratory tests, incorporating normal ranges of test results, and providing a more meaningful summary of patient data by conducting data analytics on the data.

### Comparison With Previous Work

As indicated in the Introduction section, a number of mobile PHR apps exist, such as MyChart, MyHealthKeeper, and My HealtheVet; however, these were mainly created by hospitals and EHR vendors. As a result, these PHRs obtain patient health data primarily from the corresponding EHR systems. In addition, the major purpose of these PHRs is to make some preselected patient data items available to patients cared for by the corresponding hospitals or with records in the EHR systems. These mobile PHR apps have many useful functions; for instance, they make laboratory test results easily accessible to patients, allow patients to making appointments with their doctors, and provide a platform for secure messaging between patients and their doctors [20-22,58]. However, if a person does not have an existing account with those hospitals or the specific EHR system, he/she cannot use those mobile apps. More importantly, these existing mobile PHR apps only allow patients to enter a very limited number of data items, such as blood pressure and glucose level, which makes it difficult for patients to manage other health data that may be collected from many sources, such as wearable sensors, Web-based screening tools, and self-assessment mobile apps. In other words, these existing mobile PHR apps lack the flexibility for managing various types of patient-generated data, and therefore, they cannot fill the information gap between typical clinical visits.

It is true that on both iOS and Android systems, there is a Health app (Apple Health and Samsung Galaxy Health) in which users can enter various types of patient-generated data and test results. The Health apps also offer an option to access the user’s other mHealth apps so that collect data can be shared among apps. Therefore, if the user has both this Health app and multiple mHealth apps, the user will be able to manage many data items included in this new mobile PHR. However, if the user does not have those mHealth apps, the user can only use this Health app to manage some patient-generated data and test results, such as blood pressure and number of steps. Even for these patient-generated data, these mobile operating system–specific Health apps have one major drawback, that is, the entered data will become inaccessible if the user wants to switch to a different mobile operating system. This new mobile PHR app, PittPHR, is cross-platform. In other words, the stored patient data can be accessed by any mobile device on any major mobile operating systems.

This new mobile PHR app offers a more flexible and comprehensive alternative. It is a standalone app, and it is not required to be associated with any specific EHR systems. Therefore, it has the flexibility to manage any type of health data desired by patients, instead of being limited by the rules and regulations determined by hospitals and health care providers. Patients have full control over the data managed by the app. Moreover, anyone can use this app—users do not need to have an existing account in an EHR system before they can use this app. One disadvantage is that patients have to enter all their health data manually. Although we have intentionally made the data input pages simple, it still can be burdensome when patients have to enter a specific type of data the first time or when patients have a lot of health records to manage. However, once the existing records are entered into the app, it is easy to update or enter new data items. In the future, we will consider ways to make some data input easier, for instance, making it possible for patients to scan a barcode or QR (quick response) code for items such as food, drink, and medications, a feature suggested by some of the usability study participants.

### Limitations and Future Work

A current limitation to this project is the lack of a Web portal for sharing the patient-entered information with health care providers. Currently, to share the data with providers, patients have to physically bring their mobile devices to their providers to show the information to them. In the next phase of this project, a Web portal will be created to make the PGHD readily available to providers.

The current version of this app did not help users to determine whether the entered data are normal or not in most cases. Users need to infer that from the data they obtain from their health care providers. In the next version of the app, databases and decision rules will be created and incorporated into the app to help users determine the normality of the laboratory and diagnostic test results. These databases and decision rules will help us to improve the accuracy of user-entered health data as they will determine which values are allowed for a specific test. After extensive PGHD are entered in the mobile app, it will even be feasible to make predictions and give personalized recommendations to patients, which may be helpful for improving health care quality [20,37,59-62].

The current version of the mobile PHR app simply helps users to collect and manage various types of health data. It does not provide any reminders or alerts, which is desired by some study participants in the usability study. Reminders and alerts can be very useful for some users, for instance, elderly people, cognitively impaired patients, and people with very busy schedules. However, it can be annoying for some users as well if many reminders and alerts are generated according to a schedule. Those reminders and alerts may interrupt things they are doing or pop highly sensitive information up on the screen of their mobile device while they are surrounded by a group of people, which may be a violation of the user’s privacy [63]. In the future, alerts and reminders for highly important issues may be implemented in the app, for instance, to remind users to take medications on time and at the correct dosage.

In the questionnaire study for needs assessment, there was only a small number of participants (15/609, 2.5%) with high school or lower education. Therefore, the results obtained in this study may not reflect the opinion of people with high school or lower education. The reason for this bias is probably because the questionnaire was distributed via an email, and the primary communication method for people with lower education may not be email. To recruit people with lower education into this study, other approaches, such as mailers, text messages, and posted flyers, may need to be used. The questionnaire itself may also need to be administered in a paper-based format.

The self-assessed health status of participants in the questionnaire study is also a limitation of this study. More than 90% of the study participants (550/609, 90.3%) in the questionnaire study reported that they had good, very good, or excellent health. Therefore, the needs and preferences identified in the study were only for people with at least good health status. The results could be different if the study participants were a group of people with chronic disease or some other major health issues to manage.

### Conclusions

In this project, a questionnaire was created and used to understand users’ needs, preferences, and expectations with respect to a proposed mobile PHR app. An mHealth app (PittPHR) was created according to the needs assessment results using a user-centered approach. A usability study on the app indicated that potential users were satisfied with the implementation of PittPHR and would like to use PittPHR in their personal health data management. The flexibility and customizability of this mHealth app may facilitate better personal health data management and fill the information gap between clinical visits. Further development will be conducted on this mobile app to allow it to serve users better. This mobile PHR app may help users to become more involved in their own health care and eventually improve the quality of the health care.

## Abbreviations

AES: Advanced Encryption Standard
API: application programming interface
EHR: electronic health record
HITECH: Health Information Technology for Economic and Clinical Health
ISO: International Organization for Standardization
JSON: JavaScript Object Notation
mHealth: mobile health
NIDILRR: National Institute on Disability, Independent Living, and Rehabilitation Research
ONC: Office of the National Coordinator for Health Information Technology
PGHD: patient-generated health data
PHR: personal health record
PSSUQ: Post-Study System Usability Questionnaire
QR: quick response
REST: REpresentational State Transfer
